# Colonization, growth-promoting effect, control efficacy against tobacco black shank, and mechanism of action of the tobacco endophyte *Microbacterium foliorum* 5SY32

**DOI:** 10.3389/fmicb.2026.1887697

**Published:** 2026-10-08

**Authors:** Wei Zheng, Haibang Du, Dongling Wu, Jianqiang Xu, Yebin Kang

**Affiliations:** 1 College of Horticulture and Plant Protection, Henan University of Science and Technology, Luoyang, Henan, China; 2 Luoyang Branch of Henan Provincial Tobacco Company, Luoyang, Henan, China

**Keywords:** biocontrol efficacy, colonization, endophytic bacteria, growth-promoting effect, *Microbacterium foliorum*, tobacco blank shank, transcriptome

## Abstract

Tobacco black shank (TBS) severely restricts sustainable tobacco cultivation. Endophytic bacteria serve as promising biocontrol agents due to strong host colonization ability. *Microbacterium foliorum* 5SY32, isolated from healthy tobacco, inhibits *Phytophthora nicotianae* growth by over 60% and synthesizes 15.13 μg/mL indole-3-acetic acid. This study assessed its colonization, growth-promoting capability and disease suppression efficacy via greenhouse and field trials, and explored functional mechanisms through transcriptome sequencing. Re-isolation and molecular verification proved that strain 5SY32 stably colonizes tobacco roots, stems, and leaves, with the highest population detected in roots. The strain significantly optimized root morphology and physiological activity, and improved aboveground agronomic traits, leaf chlorophyll and nitrogen content. Field yield was elevated by more than 20%. It achieved 58.58% protective and 30.31% curative efficacy in the greenhouse, and sustained 48.99%−52.31% control efficacy in the field against TBS. 5SY32 boosted antioxidant and defense enzyme activities, and activated key genes related to jasmonic acid, ethylene and abscisic acid pathways. A total of 12,485 differentially expressed genes participating in cell wall formation, oxidative response and secondary metabolism were modulated, ultimately inducing systemic resistance in tobacco. This study first clarifies the integrated mechanism of colonization, growth promotion and disease resistance induction mediated by *M. foliorum* 5SY32, offering a valuable microbial resource for green management of TBS.

## 1 Introduction

Tobacco black shank (TBS) was a devastating soil-borne disease, with the oomycete *Phytophthora nicotianae* being its sole causative agent. The disease severely restricts global tobacco production and results in substantial yield reduction and quality deterioration in tobacco crops (Guo et al., 2020). Conventional prevention and management rely heavily on chemical fungicides; nevertheless, the overuse of oomyceticides has triggered a series of ecological and agricultural problems, including environmental contamination, the emergence of pesticide-resistant pathogens, and potential food safety risks (Yan et al., 2023). These accumulating problems have created an urgent need to develop sustainable and environmentally friendly biocontrol alternatives.

Plant-microbe interactions are critical for sustaining plant health. Inoculation with single functional microbial strains can efficiently inhibit the viability of phytopathogens, further verifying the indispensable biological functions of beneficial microorganisms in plant disease resistance (Liu et al., 2012). These beneficial microbes can modulate plant physiological metabolism and trigger host immune responses, making them promising biocontrol candidates for plant disease management (Shi et al., 2024; Wang et al., 2021). Endophytic bacteria are a specific group of bacteria that asymptomatically colonize internal plant tissues and form benign symbiotic relationships with their hosts (Wang et al., 2021). As essential beneficial microbial resources, endophytic bacteria can stably reside in plant internal tissues and establish durable symbiotic interactions with host plants. The genus *Microbacterium* constitutes an important group of beneficial microorganisms, with capacities not only for environmental remediation but also for promising biocontrol potential against plant fungal and nematode diseases (Zhao et al., 2019; Patel et al., 2022). As one member of this genus, certain strains of *Microbacterium foliorum* can degrade arsenic-containing pollutants and produce cellulases, and have demonstrated excellent performance in environmental pollutant remediation and industrial enzyme production (Alka et al., 2023). Nevertheless, to date no studies have documented the isolation of endophytic *M. foliorum* from plant tissues, and its function as a plant endophyte against phytopathogens remains poorly understood. Moreover, all previously characterized *M. foliorum* strains have only been evaluated under laboratory and greenhouse experimental setups for agricultural usage. Critical knowledge gaps still exist regarding both the field performance and the underlying molecular regulatory mechanisms of *M. foliorum*, which have not yet been systematically elucidated.

In 2023, an endophytic bacterium was isolated from the upper leaves of healthy tobacco plant (cultivar Y2001). The plant sample was collected from Wangcun Village, Xiaojie Township, Luoning County, Luoyang City, Henan Province of China, with the precise geographic coordinates of 34.44°N, 111.65°E and an altitude of 533.8 m. The isolated strain exhibited an inhibitory rate of more than 60% against *P. nicotianae* and produced 15.13 μg/mL 3-indoleacetic acid (IAA). Based on phenotypic biochemical characteristics and molecular sequence alignment, the strain was taxonomically identified as *M. foliorum* and designated as 5SY32 (Du et al., 2026). Strain 5SY32 was selected from a total of 253 endophytic bacterial isolates obtained from the same tobacco field site through a two-step screening process. In the primary screening using the plate dual culture assay, isolates showing an inhibition rate >60% against *P. nicotianae* were selected. These candidates were then subjected to secondary screening based on two criteria: (1) antifungal activity of the cell-free fermentation broth (>60% inhibition), and (2) IAA production (> 5 μg/mL). Strain 5SY32 was the only isolate that met all three criteria, with an IAA yield of 15.13 μg/mL, and was thus selected for further investigation. To evaluate the field application potential of the strain 5SY32, its growth-promoting performance and biocontrol efficacy against TBS disease were investigated on tobacco plants under greenhouse and field conditions. Furthermore, transcriptomics technology was applied to unravel the underlying mechanisms responsible for its growth-promoting and disease-resistance functions. This study represents the first comprehensive report on the biocontrol potential of *M. foliorum* in tobacco cultivation, providing a promising microbial resource for the green biological control of soil-borne tobacco diseases.

## 2 Materials and methods

### 2.1 Strains, tobacco cultivar, culture media, and microbial formulation

5SY32 was preserved as glycerol stock and stored in an ultra-low temperature refrigerator. The diseased plant with typical symptoms of TBS were also collected from tobacco-growing fields in Wangcun Village, Luoning County in 2023. After diseased tobacco plants were transported to the laboratory, the pathogen was separated via tissue isolation method. The obtained pure cultures were subjected to morphological observation and molecular identification, which confirmed the pathogen as *P. nicotianae*. All the strains were deposited in the Laboratory of Molecular Identification and Green Control of Plant Diseases, Henan University of Science and Technology.

Tobacco cultivar Xibu Zhongyan 100, which shows susceptibility to TBS, was provided by Luoyang Branch of Henan Provincial Tobacco Company (Henan, China).

Luria-Bertani (LB) medium was used for bacterial cultivation in this study and potato dextrose agar medium was used to cultivate *P. nicotianae*. All media components and reagents were purchased from Aoke Chemical & Glass Instrument Sales Department (Luolong District, Luoyang, Henan, China).A commercial encapsulated granule (CG) formulation of *Bacillus subtilis* (viable count ≥ 1.0 × 10^8^ CFU/g) was purchased from Chengdu TePu Biotechnology Co., Ltd.(Chengdu, China)

### 2.2 Determination of colonization capacity of 5SY32 in tobacco plants

To evaluate the colonization ability of 5SY32, a greenhouse colonization experiment was conducted. Tobacco seedlings were cultivated in a greenhouse at the College of Horticulture and Plant Protection, Henan University of Science and Technology, under controlled conditions of 25 ± 2 °C with a 16 h light/8 h dark photoperiod. At the transplanting stage, uniformly growing and healthy seedlings were selected and transplanted into seedling trays (54 × 28 × 10 cm) filled with sterilized cultivation substrate (total organic matter ≥ 50%; Shenxian Yinong Seedling Substrate Co., Ltd. Shan Dong, China). After seedling acclimatization, each seedling was root-irrigated with 10 mL of 5SY32 bacterial suspension (1 × 10^8^ CFU/mL). This concentration was selected based on previous studies using root irrigation with beneficial endophytic bacteria for biocontrol and plant growth-promotion assays (Zhu et al., 2026). Equal volumes of sterile water and LB medium were applied as blank and vehicle controls, respectively. All treatments were maintained under uniform management conditions. The experiment was arranged in a completely randomized design, with 15 seedlings per treatment and three biological replicates, repeated twice independently.

On the 21st day post-treatment, root, stem, and leaf tissues were harvested from tobacco seedlings in each treatment group for qualitative and quantitative analysis of colonization.

Qualitative detection was carried out by tissue isolation followed by colony PCR verification. Referring to the method of Sánchez-Campos et al. (2013), surface-sterilized tissue homogenates were spread onto LB agar plates and incubated. Suspected colonies with morphological characteristics identical to those of strain 5SY32 were selected and subjected to colony PCR for molecular confirmation (Packeiser et al., 2013). Based on the *gyrB* gene sequence of strain 5SY32, a pair of specific primers was designed: D1F (5′-GTAGAGGTGAAGCAGAAGGG-3′) and D1R (5′-GAGCCGAAGGCCTGGATCAT-3′), yielding an expected amplicon size of 870 bp. The 25 μL PCR reaction mixture contained 12.5 μL of 2 × Taq PCR Master Mix, 1 μL of each primer (10 μmol/L), 1 μL of DNA template (100 ng/μL), and ddH_2_O to a final volume of 25 μL. The thermal cycling program was set as follows: initial denaturation at 94 °C for 4 min; 30 cycles of 94 °C for 30 s, 56 °C for 30 s, and 72 °C for 90 s; and a final extension at 72 °C for 10 min. The amplified products were sequenced and subjected to BLAST analysis against the NCBI database to confirm strain identity.

Quantitative detection was performed using the dilution plate counting method. On the 21st day post-treatment, tobacco root, stem, and leaf tissues were aseptically collected and 2 g of each tissue was weighed and thoroughly ground in 2 mL of sterile water to obtain a homogenate. The tissue homogenates were serially diluted to 10^−4^, and 100 μL of an appropriate dilution was spread onto LB agar plates. After incubation at 28 °C until colonies were clearly visible, colonies displaying the typical morphology of 5SY32 were enumerated. The number of culturable endophytic bacteria per gram of tissue was calculated based on the dilution factor, and colonization density was expressed as CFU/g fresh weight (Zhu et al., 2026).

### 2.3 Effects of 5SY32 on agronomic traits of tobacco seedlings

Following confirmation of the colonization ability of 5SY32, a second independent greenhouse experiment was conducted under the same conditions. Each seedling was root-irrigated with 10 mL of 5SY32 suspension (1 × 10^8^ CFU/mL) (Zhu et al., 2026). Sterile water and LB medium control were included. The experiment was arranged in a completely randomized design, with 15 seedlings per treatment and three biological replicates, repeated twice independently. The greenhouse conditions were maintained at 25±2 °C, the photoperiod was 16 h of light and 8 h of darkness. On day 21 post-treatment, stem circumference, maximum leaf length, and maximum leaf width were measured in accordance with the Chinese tobacco industry standard *Methods for Investigation and Measurement of Tobacco Agronomic Traits* (YC/T 142–2010). The leaf area was calculated using the empirical formula:

Leaf area (cm^2^) = 0.6345 × Maximum leaf length (cm) × Maximum leaf width (cm).

### 2.4 Preventive efficacy of 5SY32 against tobacco black shank

Four treatments were established: sterile water, LB medium, *B. subtilis* CG (1.0 × 10^8^ CFU/g), and 5SY32. Each treatment had three biological replicates with 15 seedlings per replicate. After acclimatization, 10 mL of 5SY32 suspension (1 × 10^8^ CFU/mL) or equivalent control solutions were root-applied. A 3-day interval was allowed between bacterial treatment and pathogen inoculation. This interval was chosen to enable sufficient rhizosphere colonization and establishment of strain 5SY32, as well as to allow for the induction of plant defense responses prior to pathogen challenge, which is a common practice in biocontrol studies using beneficial rhizobacteria (Samaras et al., 2021). At 3 days post-bacterial treatment, each seedling was inoculated with 10 mL of *P. nicotianae* spore suspension (1 × 10^6^ sporangia/mL). At 21 days post-inoculation, disease severity was evaluated on each tobacco plant using the grading criteria specified in the Chinese national standard GB/T 23222–2008 (Grades 0, 1, 3, 5, 7, and 9), based on the proportion of stem circumference covered by lesions and the extent of leaf wilting, where 0- no symptoms; 1- stem lesions covering < 1/3 of stem circumference or wilting of ≤ 1/3 of leaves; 3- lesions encircling 1/3–1/2 of the stem or wilting of 1/3–1/2 of leaves; 5 = lesions extending >1/2 but not completely encircling the stem or wilting of 1/2–2/3 of leaves; 7 = complete stem girdling or wilting of >2/3 of leaves; and 9 = plants nearly dead or collapsed. Disease incidence and disease index (DI) were then calculated accordingly. The calculation formula was defined as: DI = 100 × Σ (ni × vi) / (N × V). In this formula, ni represents the number of plants at the i-th disease grade; vi indicates the score corresponding to the i-th disease grade; N is the total number of investigated plants; and V refers to the highest disease grade in the grading standard. The preventive biocontrol efficacy of 5SY32 was evaluated by comparing disease incidence and disease index among different treatment groups.

### 2.5 Therapeutic efficacy of 5SY32 against tobacco black shank

The experimental layout was consistent with the aforementioned preventive efficacy assay. Briefly, uniform tobacco seedlings were initially inoculated with 10 mL of *P. nicotianae* sporangia suspension (1 × 10^6^ sporangia/mL). On day 10 post-pathogen inoculation, obvious disease symptoms gradually appeared approximately. Subsequently, each infected seedling was treated with 10 mL of 5SY32 bacterial suspension (1 × 10^8^ CFU/mL) or an equal volume of control solution. Disease development was assessed 21 days after pathogen inoculation. Disease grading and statistical analysis was evaluated following the same standard in section 2.4. Disease incidence and disease index were calculated using the same formula described above. The therapeutic efficacy of 5SY32 was determined via comparative analysis of disease parameters between treatment and control groups.

### 2.6 Effects of 5SY32 on root activity and morphogenesis of tobacco seedlings

Tobacco root activity was determined 21 days after root irrigation with 5SY32 using the triphenyltetrazolium chloride (TTC) staining method (Kandel et al., 2017). Meanwhile, the intact root system was scanned using an Epson Expression 12000XL root scanner, and morphological parameters were further analyzed with WinRHIZO Pro image analysis software. Before scanning, root samples were placed in a transparent observation tank filled with deionized water to maintain natural morphological characteristics. Several key root morphological indicators were quantified, including total root length (TRL), total projected area (TPA), total surface area (TSA), average diameter (AD), root tip number (RTN), and fork number (FN).

### 2.7 Assay of antioxidant and defense enzyme activities and malondialdehyde level in tobacco seedling roots

21 days after 5SY32 root irrigation, tobacco root tissues were collected to determine the activities of antioxidant and defense-related enzymes. The detected enzymes included peroxidase (POD), superoxide dismutase (SOD), catalase (CAT), phenylalanine ammonia-lyase (PAL), and polyphenol oxidase (PPO). Root malondialdehyde (MDA) content was also determined. All biochemical indicators were measured using commercial detection kits (Beijing Solarbio Science & Technology Co., Ltd. Beijing, China), and all experimental operations were strictly performed in accordance with the manufacturer's protocols.

### 2.8 Growth-promoting effect of 5SY32 on field-grown tobacco plants and its biocontrol efficacy against tobacco black shank

Field trials were carried out from 1 May to 26 August 2025 at two experimental plots in Luoyang City, China: Plot A (Shicun Village, Gaocun Township, Yiyang County, Luoyang City; 34°32′N, 111°46′E; 413 m altitude; cinnamon soil) and Plot B (Wangcun Village, Xiaojie Township, Luoning County, Luoyang City; 34.44°N,111.65°E; altitude 533.8 m; cinnamon soil). The baseline physicochemical properties of the soil at Plot A were as follows: available nitrogen 69.59 mg/kg, available phosphorus 22.19 mg/kg, available potassium 283.13 mg/kg, organic matter 15.68 g/kg. At Plot B, the soil properties were: available nitrogen 63.31 mg/kg, available phosphorus 18.32 mg/kg, available potassium 268.59 mg/kg, organic matter 12.74 g/kg. The two experimental sites were located in a region characterized by a warm temperate continental monsoon climate, with four distinct seasons and concurrent rainfall and heat. Plot A had a mean annual temperature of 14.8–15.2 °C, a mean annual precipitation of 639–666 mm, an annual sunshine duration of 1,879.5–2,083.6 h, and a frost-free period of 200–228 days. For Plot B, the mean annual temperature was 13.7–13.9 °C, the mean annual precipitation was 568–606 mm, the annual sunshine duration was 2,006-2,217.6 h, and the frost-free period was 212–216 days. Precipitation at both sites was predominantly concentrated from June to September.

The experiment was arranged in a randomized complete block design (RCBD) with five blocks per site. Within each block, four treatments were randomly assigned: (1) 5SY32 application, (2) sterile water control, (3) LB medium control, and (4) *B. subtilis* CG (1.0 × 10^8^ CFU/g) as a positive biocontrol control. Each treatment consisted of 30 tobacco plants per replicate, with five biological replicates (blocks) per treatment, giving a total of 150 plants per treatment per site. Each plot measured 6 m × 4 m (24 m^2^), with 1.0 m spacing between plots and border rows planted around the experimental area to minimize edge effects. Standard agronomic practices, including irrigation, fertilization, and pest management, were applied uniformly to all plots according to local recommendations.

For the 5SY32 treatment, the bacterial suspension was diluted with sterile water at a volume ratio of 1:9 (1 L bacterial suspension mixed with 9 L sterile water). The tobacco seedlings was transplanted on 1 May, 2025, and 100 mL of the diluted bacterial suspension was subsequently root-irrigated of each tobacco plant. An equal volume of sterile water, pure LB medium, and 1.0 × 10^8^ CFU/g *B. subtilis CG* suspension was applied to the corresponding control groups, respectively. All treatments were repeatedly irrigated once again at 7 days after transplantation, with a total of two root-irrigation applications. The same experimental designs and field management regimes were applied at both plots.

Plant agronomic traits, including plant height, stem circumference, maximum leaf length, maximum leaf width, chlorophyll content, and leaf nitrogen (N) content, were determined at the rosette stages (24 June) and vigorous growth stages (20 July) in accordance with the Chinese tobacco industry standard described in section 2.3. At the harvesting stage (26 August), upper and middle leaves of tobacco plants were sampled from each treatment to record the fresh weight. All leaf samples were subjected to deactivation at 105 °C for 30 min and subsequently dried to a constant weight at 65 °C to determine dry weight and calculate tobacco yield. The relative increment rates of fresh and dry weight were calculated relative to the corresponding control groups. During the vigorous growth stage, tobacco disease severity was evaluated following the same standard in section 2.4. The disease incidence and disease index were calculated in accordance with the aforementioned standard method.

### 2.9 Transcriptomic analysis of tobacco resistance to black shank induced by 5SY32

To elucidate the induced resistance mechanism mediated by 5SY32 against TBS, three experimental treatments were set up in this study, including a sterile water control group (W), a single *P. nicotianae* inoculation group (P), and a combined treatment group (M) with 5SY32 pretreatment prior to *P. nicotianae* infection. Each treatment contained three biological replicates, and thirty tobacco seedlings were included in each replicate. All tobacco seedlings were cultivated under consistent greenhouse conditions as previously described. Specifically, each tobacco seedling in group M was root-treated with 3 mL of 5SY32 bacterial suspension at a concentration of 1 × 10^8^ CFU/mL. Three days after bacterial pretreatment, the seedlings were inoculated with *P. nicotianae* zoospore suspension (1 × 10^6^ spores/mL). For group P, seedlings were solely inoculated with an equivalent volume of the zoospore suspension, while seedlings in group W were treated with an equal volume of sterile water to serve as the blank control. Basal stem tissues of tobacco plants were harvested at 3 days post-pathogen inoculation. For each treatment, tissue samples with identical fresh weight were collected in triplicate for subsequent total RNA extraction.

Total RNA was isolated from the collected tissue samples using the TRIzol reagent method. The concentration, purity, and integrity of the extracted RNA were systematically determined, and only RNA samples with an RNA Integrity Number (RIN) ≥ 7.0 were retained for subsequent experiments. After genomic DNA removal, strand-specific RNA-seq libraries were constructed using the Illumina NEBNext Ultra II Directional RNA Library Prep Kit. Subsequently, paired-end 150 bp sequencing was performed on the Illumina NovaSeq platform to generate raw sequencing data.

Following strict quality control procedures, high-quality clean reads were mapped to the tobacco reference genome. The expression levels of annotated genes were quantified and normalized for subsequent differential expression analysis. Differentially expressed genes (DEGs) were screened and identified, and Gene Ontology (GO) and Kyoto Encyclopedia of Genes and Genomes (KEGG) enrichment analyses were further conducted to dissect the potential biological functions and metabolic pathways of these DEGs. To verify the reproducibility and reliability of the transcriptomic data, several candidate DEGs associated with plant defense responses and hormone signaling pathways were selected for quantitative real-time PCR (qPCR) validation. The qPCR results exhibited high consistency with the transcriptome sequencing data, thereby confirming the credibility and validity of the transcriptomic analysis in this study.

### 2.10 Statistical analysis

All statistical analyses were performed using SPSS Statistics (version 26.0; IBM Corp., Armonk, NY, USA). Data were first tested for normality using the Shapiro–Wilk test and for homogeneity of variances using Levene's test. When assumptions were met, differences among treatment groups were analyzed using one-way analysis of variance (ANOVA), followed by Tukey's honestly significant difference (HSD) *post hoc* test for multiple comparisons. In cases where the assumptions of ANOVA were violated, the Kruskal–Wallis non-parametric test was applied, followed by Dunn's *post hoc* test with Bonferroni correction. All reported values represent the mean ± standard deviation (SD) of three biological replicates. A significance level of α = 0.05 was used for all statistical tests, and results with *p* < 0.05 were considered statistically significant.

## 3 Results

### 3.1 Colonization of 5SY32 in tobacco plants

As displayed in Figure 1A, colonies morphologically consistent with strain 5SY32 were recovered from the roots, stems, and leaves of tobacco plants inoculated with 5SY32 suspension (1 × 10^8^ CFU/mL), while no identical colonies were observed in the untreated control group (Figure 1A). To verify the identity of the isolated colonies, colony PCR amplification was conducted. An 870 bp specific fragment was amplified from several endophytic isolates derived from tobacco roots, stems, and leaves, which matched the target band of the positive control (genomic DNA of strain 5SY32). By contrast, no non-specific amplification band was detected in the ddH_2_O negative control (Figure 1B).

**Figure 1:**
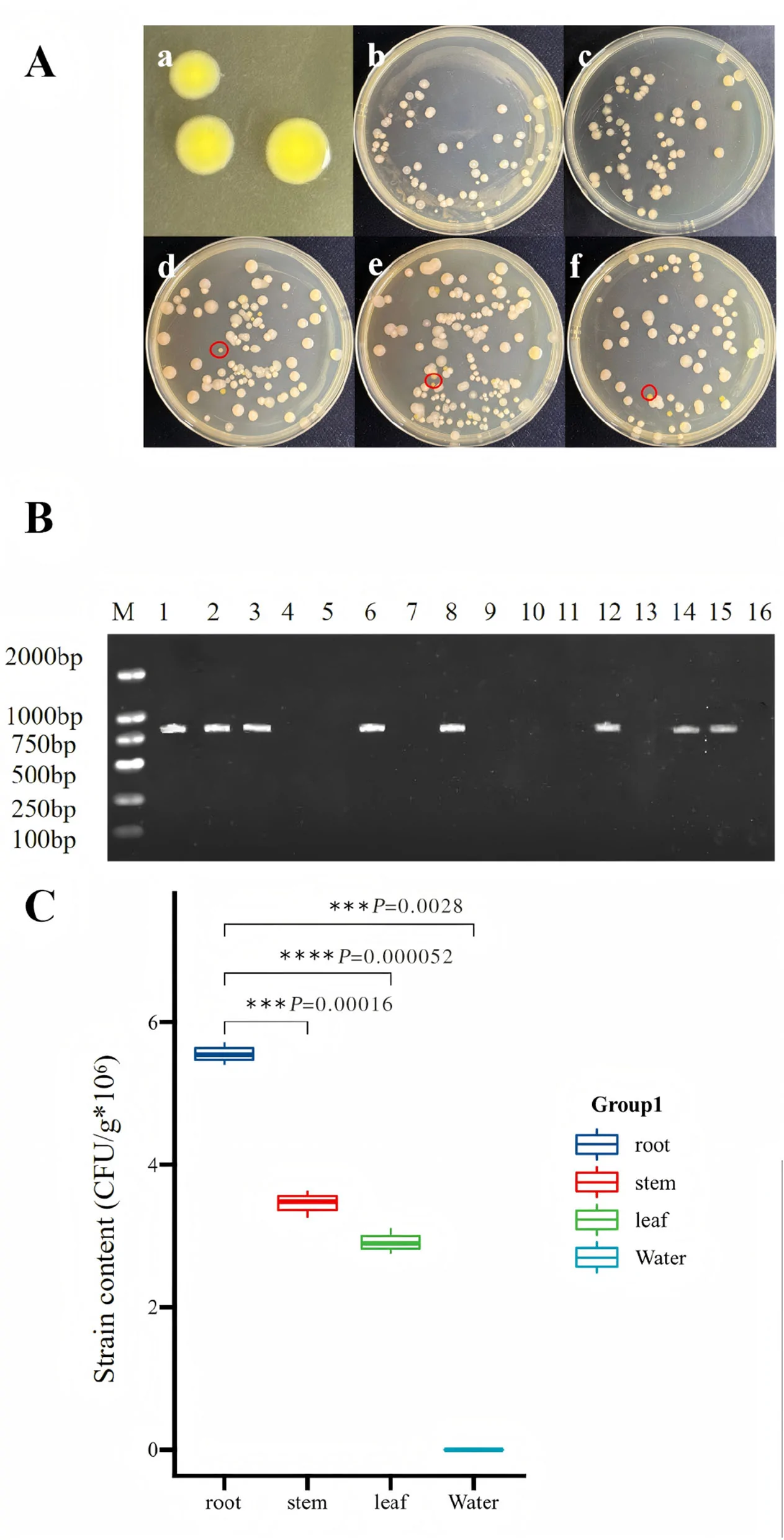
Colonization of Microbacterium phyllosphaerae 5SY32 in different tissues of tobacco plants. **(A)** Colonization of strain 5SY32 detected by bacterial colony isolation from different treatment groups.a, colonies of strain 5SY32;b, colonies isolated from tobacco seedlings treated with sterile water;c, colonies isolated from tobacco seedlings treated with LB medium;d, colonies isolated from roots of tobacco seedlings inoculated with 5SY32;e, colonies isolated from stems of tobacco seedlings inoculated with 5SY32;f, colonies isolated from leaves of tobacco seedlings inoculated with 5SY32. **(B)** Colonization of strain 5SY32 detected by *gyrB* gene-targeted PCR.M, DL2000 DNA marker;1, genomic DNA of strain 5SY32 (positive control);2–5, leaf tissues treated with 5SY32;6–10, stem tissues treated with 5SY32;11–15, root tissues treated with 5SY32;16, ddH_2_O (negative control). **(C)** Distribution of *Microbacterium phyllosphaerae* 5SY32 in different tissues of tobacco plants. Statistical significance among different treatments is indicated by asterisks: ***P < 0.001, ****P < 0.0001; ns, not significant.

Quantitative colonization analysis further revealed distinct tissue-specific colonization patterns of strain 5SY32 in tobacco (Figure 1C). The colonization abundance was the highest in roots [(5.55 ± 0.16) × 10^6^ CFU/g], followed by stems [(3.45 ± 0.19) × 10^6^ CFU/g], and the lowest in leaves [(2.92 ± 0.18) × 10^6^ CFU/g]. It should be noted that the population data presented here represent recoverable culturable cells, which may underestimate the total bacterial population present in planta.

### 3.2 Effects of 5SY32 on agronomic and physiological traits of tobacco seedlings

The leaf length, leaf width, stem circumference, and chlorophyll content were significantly higher in the 5SY32 treatment group than in all control groups (Figure 2). Compared with the sterile water control, 5SY32 treatment increased maximum leaf length, leaf width, stem circumference, chlorophyll content, and nitrogen content by 38.24%, 28.16%, 15.34%, 40.14%, and 6.43%, respectively. Relative to the LB treatment, the above indicators increased by 9.31%, 19.79%, 6.03%, 16.94%, and 2.52%, respectively (Table 1). These data confirm that 5SY32 exerts a prominent growth-promoting effect on the aboveground tissues of tobacco seedlings.

**Figure 2:**
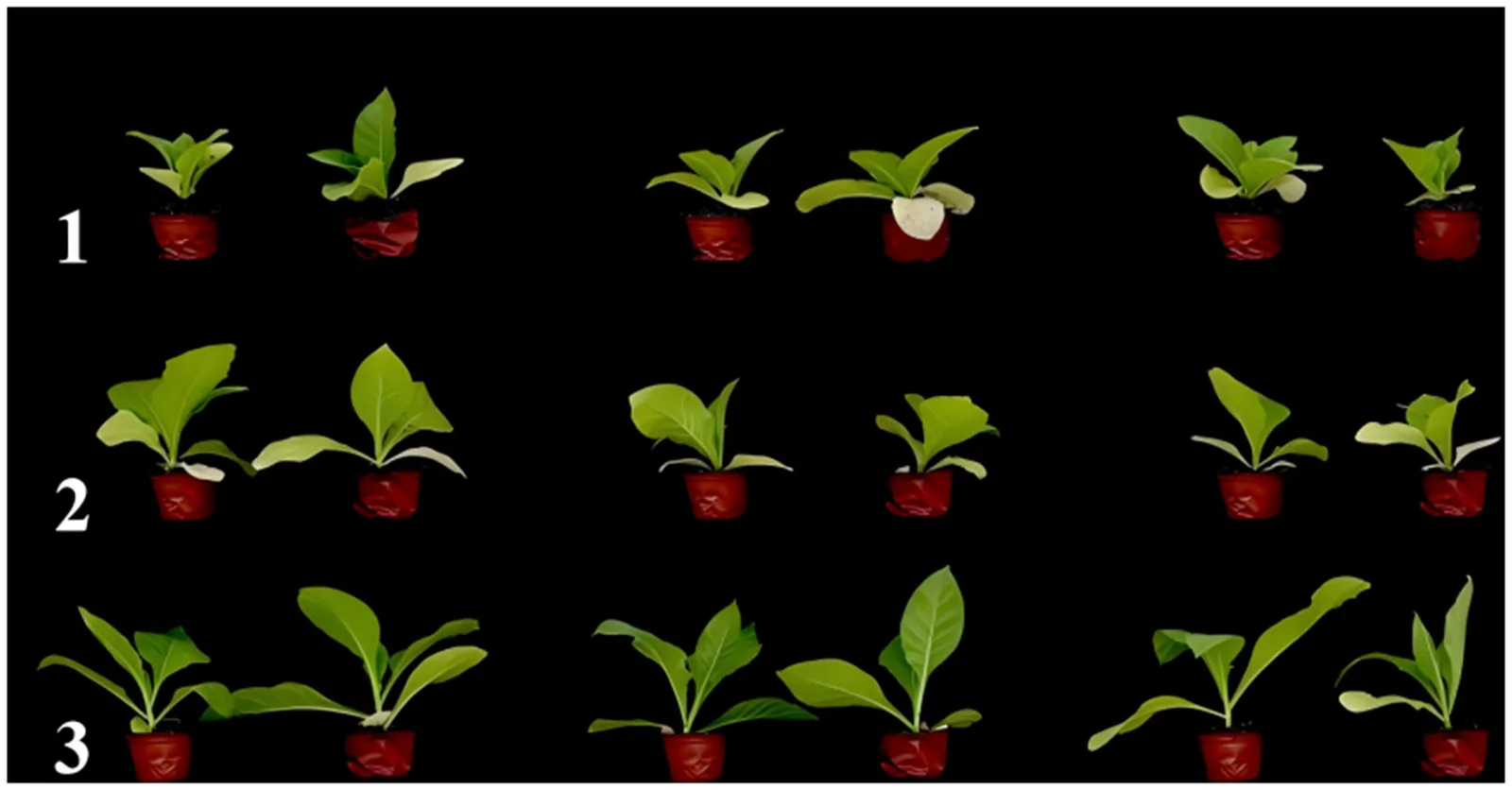
Effects of strain 5SY32 on the growth of tobacco seedlings. 1, fresh water; 2, LB medium; 3, 5SY32 fermentation suspension.

**Table 1:** Effects of 5SY32 on the growth of tobacco seedlings.

Treatment	Maximum leaf length (cm)	Maximum leaf width (cm)	Stem circumference (mm)	Chlorophyll content (SPAD)	Nitrogen content (mg/g)
Water	20.66 ± 1.60 c	9.73 ± 0.96 c	23.79 ± 0.64 c	18.09 ± 0.15 c	9.18 ± 1.08 b
LB	26.11 ± 0.60 b	10.41 ± 2.78 b	25.88 ± 0.77 b	21.67 ± 0.83 b	9.53 ± 0.74 ab
5SY32	28.54 ± 0.96 a	12.47 ± 1.12 a	27.44 ± 0.68 a	25.34 ± 1.27 a	9.77 ± 1.23 a

Data are presented as mean ± SD (*n* = 3 biologically independent replicates). Different lowercase letters indicate significant differences among tissues (one-way ANOVA followed by Tukey's HSD test, *P* < 0.05).

### 3.3 Greenhouse control efficacy of 5SY32 against tobacco black shank

In the preventive assay, the root irrigation treatment with 5SY32 achieved a disease control efficacy of 58.58%, which was slightly higher than that of 1.0 × 10^8^ CFU/g *B. subtilis* CG (57.92%); (Table 2). In the therapeutic assay, the control efficacy of 5SY32 reached 30.31%, also exceeding that of 1.0 × 10^8^ CFU/g *B. subtilis* CG (26.26%). These results suggest that 5SY32 possesses both preventive and therapeutic potential against TBS, with an overall biocontrol efficacy superior to 1.0 × 10^8^ CFU/g *B. subtilis* CG. Notably, the preventive efficacy (58.58%) was approximately 1.93-fold higher than the therapeutic efficacy (30.31%) of the same strain. This marked difference (28.27 percentage points) may be attributed to the fact that *M. foliorum* 5SY32 acts primarily as a protective biocontrol agent, possibly via root colonization and competition, rather than via direct suppression of established infections. However, the moderate therapeutic efficacy (30.31%) still suggests that the strain produces diffusible antifungal metabolites that can partially suppress disease progression even after infection.

**Table 2:** Protective and curative efficacies of 5SY32 against tobacco black shank.

Treatment	Protective action		Therapeutic action	
	Disease index	Control effect/(%)	Disease index	Control effect/(%)
Water	74.81 ± 2.57 a	–	74.81 ± 2.57 a	–
LB	73.33 ± 4.45 a	–	73.33 ± 4.45 a	–
B.S	30.86 ± 4.27 b	57.92	54.07 ± 11.18 b	26.26
5SY32	30.37 ± 7.81 b	58.58	51.11 ± 4.45 b	30.31

Data are presented as mean ± SD (*n* = 3 biologically independent replicates). Different lowercase letters indicate significant differences among treatments (one-way ANOVA, Tukey's HSD, *P* < 0.05).

### 3.4 5SY32 facilitates root activity and morphogenesis in tobacco seedlings

Tobacco plants in the 5SY32 treatment group exhibited markedly higher root activity than those in the water and LB groups, with increases of 184.49% and 47.93%, respectively (Figures 3A, B). 5SY32 substantially facilitated the root morphological development of tobacco seedlings. Specifically, multiple key root morphological indicators, including TRL, TPA, TSA, RTN, and root FN, were significantly elevated in the 5SY32 treatment relative to other groups. Compared with the sterile water control, 5SY32 treatment increased the above five indicators by 70.6%, 62.7%, 40.2%, 50.2%, and 43.2%, respectively. In comparison with the LB treatment, the corresponding increments were 55.4%, 46.3%, 31.9%, 39.4%, and 29.8% (Figures 3C, D). Collectively, these findings demonstrate that 5SY32 can effectively optimize root morphology and promote root branching of tobacco plants.

**Figure 3:**
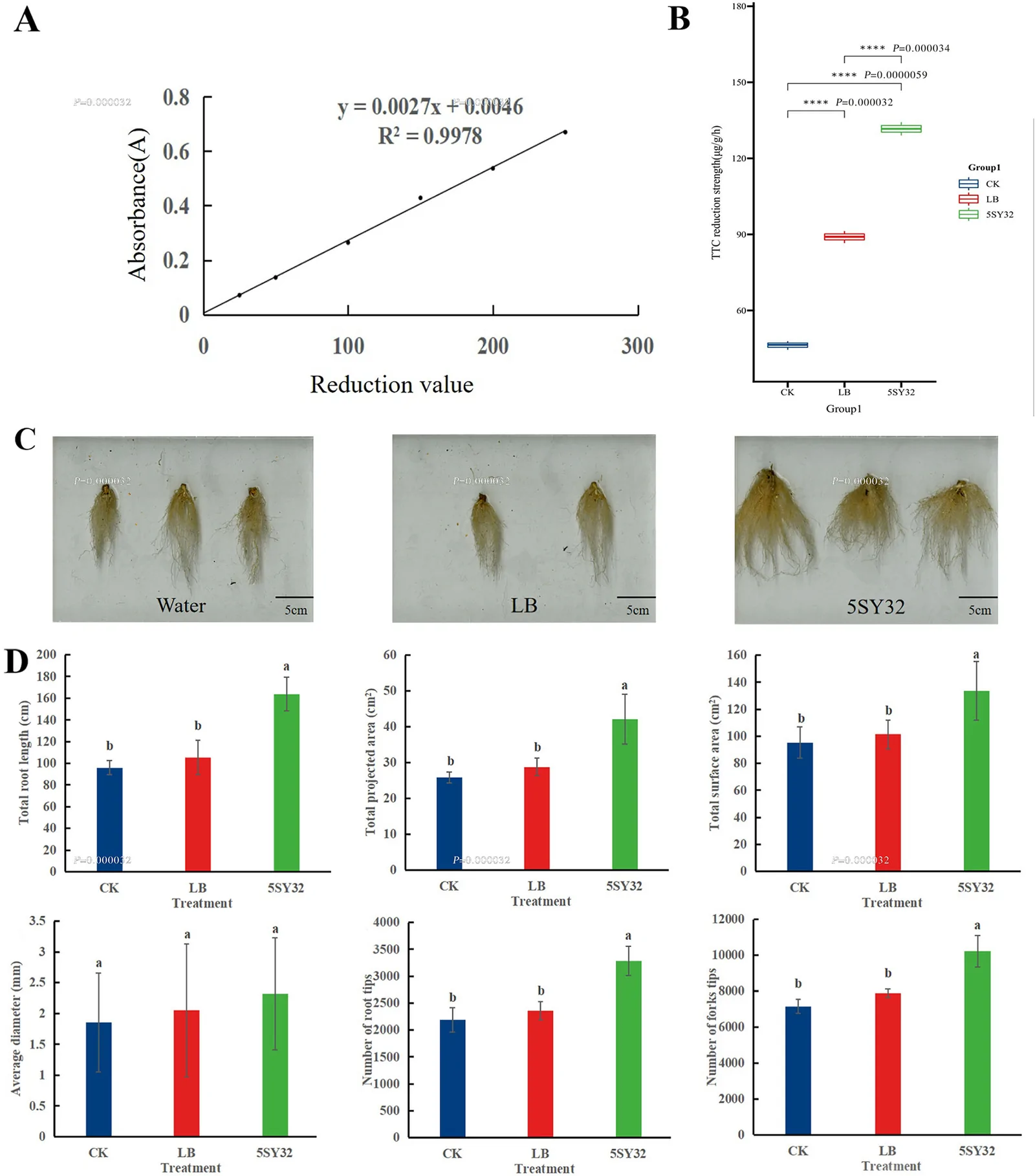
Effects of 5SY32 strain on root activity and morphological development of tobacco plants. **(A)** Standard curve of TTC reduction for quantification of triphenylformazan (TTF). **(B)** Quantitative determination of root activity using the TTC reduction method. **(C)** Representative images of tobacco root morphology under different treatments. **(D)** Effects of 5SY32 on root morphological traits of tobacco seedlings. Statistical significance among different treatments is indicated by asterisks: ^****^P < 0.0001; ns, not significant.

### 3.5 Effects of 5SY32 on antioxidant and defense enzyme activities and malondialdehyde content in tobacco seedling roots

5SY32 could significantly improve the activities of antioxidant and defense enzymes, including SOD, POD, CAT, PAL, and PPO, in comparison with all control groups (Figure 4). The SOD activity of tobacco roots in the 5SY32 group reached 904.57 U/g, which was remarkably higher than that in the sterile water control (352.49 U/g). Moreover, the POD (20,281.50 U/g), CAT (53.42 U/g), PAL (21.51 U/g) and PPO (67.20 U/g) activities were also significantly superior to those in other treatment groups. The coordinated enhancement of antioxidant enzymes (SOD, POD, and CAT) and key enzymes involved in the phenylpropanoid metabolic pathway (PAL and PPO) indicated that 5SY32 could alleviate oxidative stress in tobacco seedlings by inducing systemic resistance, thereby improving plant defense capability and growth status. Tobacco seedlings treated with 5SY32 exhibited distinctly higher MDA levels relative to all controls, possibly resulting from physiological alterations occurring during strain-triggered defense activation.

**Figure 4:**
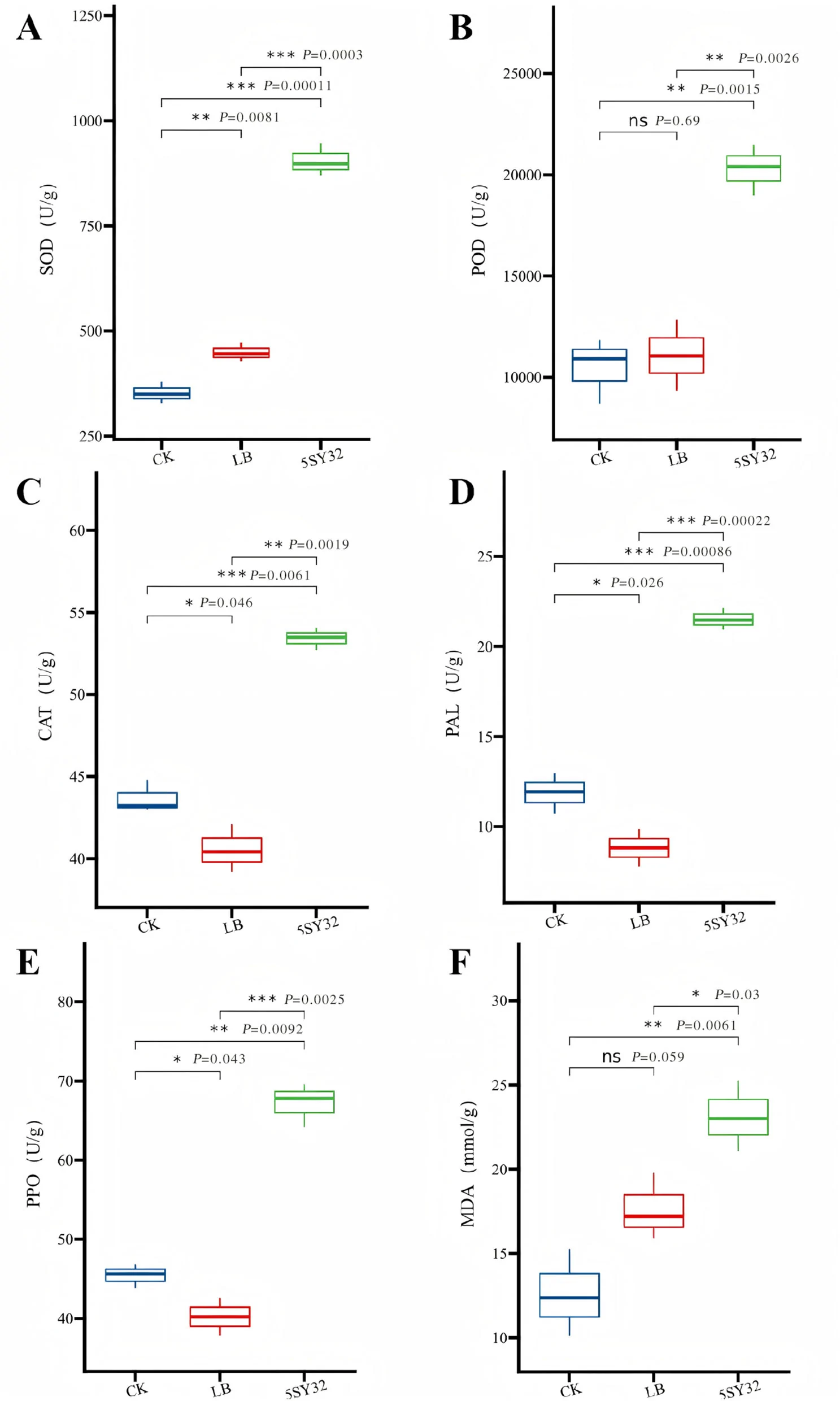
Effects of 5SY32 on defense enzyme activities in tobacco seedlings. **(A–F)** Activities of defense-related enzymes in tobacco roots: **(A)** SOD activity; **(B)** POD activity; **(C)** CAT activity; **(D)** PAL activity; **(E)** PPO activity; and **(F)** MDA activity. Statistical significance among different treatments is indicated by asterisks: *P < 0.05, **P < 0.01, ***P < 0.001; ns, not significant.

### 3.6 Growth promotion and biocontrol performance of 5SY32 under field conditions

Field survey schedules post tobacco seedling transplanting were presented in Figure 5A. At the rosette stage (T1), the 5SY32 treatment group exhibited significantly higher maximum leaf length, maximum leaf width, stem circumference, and plant height than the sterile water and LB control groups. At the vigorous growth stage (T2), all measured agronomic traits in the 5SY32 group were markedly higher than those in the sterile water and LB groups (Figure 5B). Phenotypic observations showed that tobacco plants treated with 5SY32 presented dark green leaves, orderly leaf arrangement, compact canopy structure, and stronger growth vigor (Figure 5C).

**Figure 5:**
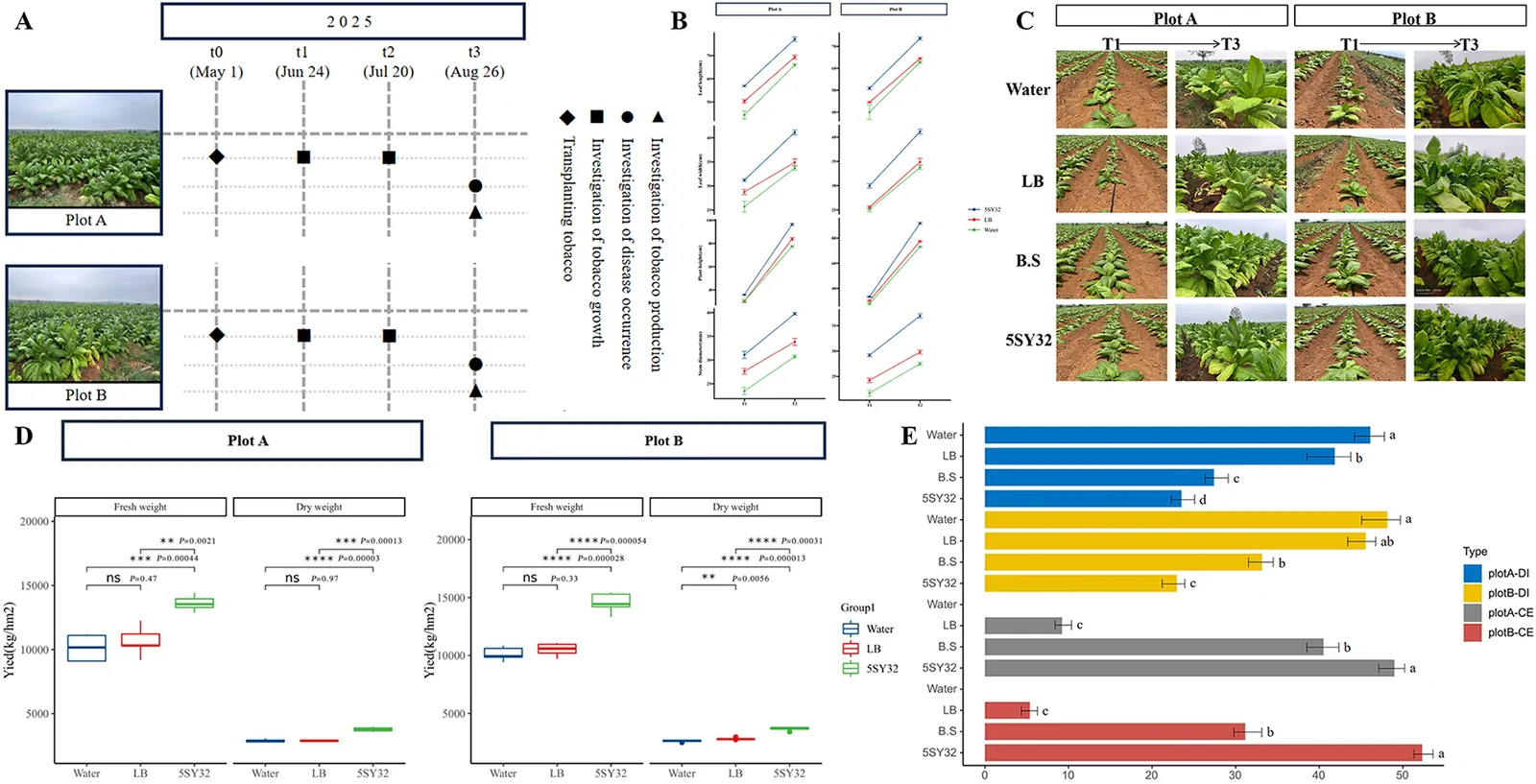
Field control effect of 5SY32 strain on tobacco black shank. **(A)** Schematic diagram of the field experimental design. Experiments were performed at two sites (Plot A and Plot B) with similar environmental conditions. Sampling and measurement were conducted at three time points (T1, T2, T3). **(B)** Plant growth parameters, including leaf length, leaf width, plant height, and stem diameter, measured at T1 and T2 for each treatment in Plot A and Plot B. **(C)** Representative phenotypes of tobacco plants under different treatments at T1 and T3 in both plots. **(D)** Fresh weight and dry weight of middle tobacco leaves sampled at T3 in Plot A and Plot B. Box plots show the median and interquartile range (*n* = 3). **(E)** Disease index (DI) and control efficacy (%) of each treatment at T3. Error bars represent the standard deviation of three biological replicates. Data are presented as mean ± standard deviation (SD). Statistical analysis was performed using DPS v9.01 software by one-way analysis of variance (ANOVA) followed by Tukey's test (*p* < 0.05). Statistical significance: **p < 0.01; ***p < 0.001; ****p < 0.0001; ns, not significant. Different lowercase letters indicate significant differences among treatments (*p* < 0.05).

The biomass determination results demonstrated that the fresh and dry weights of tobacco plants in the 5SY32 treatment were significantly higher than those in the sterile water and LB groups in both experimental plots (Figure 5D). Specifically, the fresh weight of tobacco in Plot A was 13,617.84 kg/hm^2^ in the 5SY32 treatment group, compared to 10127.04 kg/hm^2^ in the sterile water control, representing a 34.47% increase. In Plot B, the fresh weight was 14,541.95 kg/hm^2^ in the treatment group vs. 10136.95 kg/hm^2^ in the control, representing a 43.47% increase. Similar trends were observed for dry weight.

Furthermore, 5SY32 effectively reduced the incidence of TBS under field conditions. The biocontrol efficacies of 5SY32 in Plot A and Plot B were 48.99% and 52.31%, respectively, which were higher than those of 1.0 × 10^8^ CFU/g *B. subtilis* CG (19.15% and 18.04%, respectively) (Figure 5E). In summary, 5SY32 has stable and excellent biocontrol performance against TBS, showing obvious advantages over commercial microbial agents.

### 3.7 Transcriptomic analysis of growth promotion and disease resistance induced by 5SY32

To dissect the molecular mechanisms underlying 5SY32-mediated growth promotion and induced resistance against TBS, transcriptome sequencing was performed on tobacco seedlings. Plant samples were harvested when obvious disease symptoms appeared in the pathogen-inoculated control group (P). Each sample generated approximately 6 GB of high-quality clean data, with an average Q30 value of 93.81% and a genome mapping rate above 96.47% (Table 3). These metrics confirmed reliable and high-quality transcriptome sequencing data for subsequent bioinformatic analysis.

**Table 3:** Quality assessment of transcriptome sequencing data.

Sample	Raw Reads	GC (%)	N (%)	(%)	(%)	Clean Reads	Clean Reads (%)	Mapped Reads	Mappped Reads (%)
M_1	36627216	42.50	0.003003	98.28	93.07	35960514	98.18	34926155	97.12
M_2	51054488	42.70	0.005091	98.64	94.27	50349438	98.62	48597181	96.52
M_3	37581450	42.74	0.001636	98.52	93.61	37071838	98.64	35879922	96.78
W_1	37320424	42.35	0.003036	98.38	93.34	36698518	98.33	35061828	95.54
W_2	36776896	41.94	0.002973	98.38	93.23	36218976	98.48	35204800	97.20
W_3	62544580	42.17	0.006978	98.79	94.94	61702894	98.65	59447874	96.35
P_1	54997694	42.45	0.006009	98.70	94.51	54262484	98.66	52495547	96.74
P_2	35966554	42.37	0.003013	98.32	93.10	35375930	98.36	34485643	97.48
P_3	50545466	42.70	0.005233	98.62	94.23	49804590	98.53	48501743	97.38

Sample: Sample Name; Raw Reads: Total Raw Reads; GC (%): percentage of bases that are guanine (G) or cytosine (C); N (%): percentage of ambiguous bases (represented as “N”); Q20 (%): percentage of bases with a base-calling accuracy of ≥ 99% (i.e., Phred quality score ≥ Q20); Q30 (%): percentage of bases with a base-calling accuracy of ≥ 99.9% (i.e., Phred quality score ≥ Q30); Clean Reads: high-quality reads obtained after removing raw reads with3′ adapter contamination and those with an average quality score below Q20; Clean Reads (%): percentage of high-quality reads relative to total raw sequencing reads; Mapped Reads: total number of reads that align to the reference genome; Mapped Reads (%): percentage of Mapped Reads that are mapped to the Clean Reads.

Transcriptomic profiling revealed pronounced transcriptional variations in tobacco plants under different treatments upon 5SY32 inoculation. In the comparison of W vs. M, a total of 12,485 DEGs were screened based on the thresholds of |log2FC| ≥ 1 and FDR < 0.05, consisting of 6,579 upregulated and 6,906 downregulated genes. Volcano plot visualization (Figure 6A) demonstrated that 5SY32 treatment triggered widespread transcriptional alterations relative to the water control, indicating a substantial modulatory effect of 5SY32 on the global transcriptome of tobacco. For the P vs. M comparison, 10,178 DEGs were identified, including 4,790 upregulated and 5,388 downregulated genes. The lower number of DEGs in the P vs. M group than in the W vs. M group suggested distinct transcriptional patterns induced by 5SY32 with and without pathogen challenge. Additionally, numerous DEGs were detected in the W vs. P comparison, with a marked increase in upregulated genes accompanied by abundant downregulated genes, implying that pathogen infection induced extensive transcriptional reprogramming in tobacco (Figures 6A–C).

**Figure 6:**
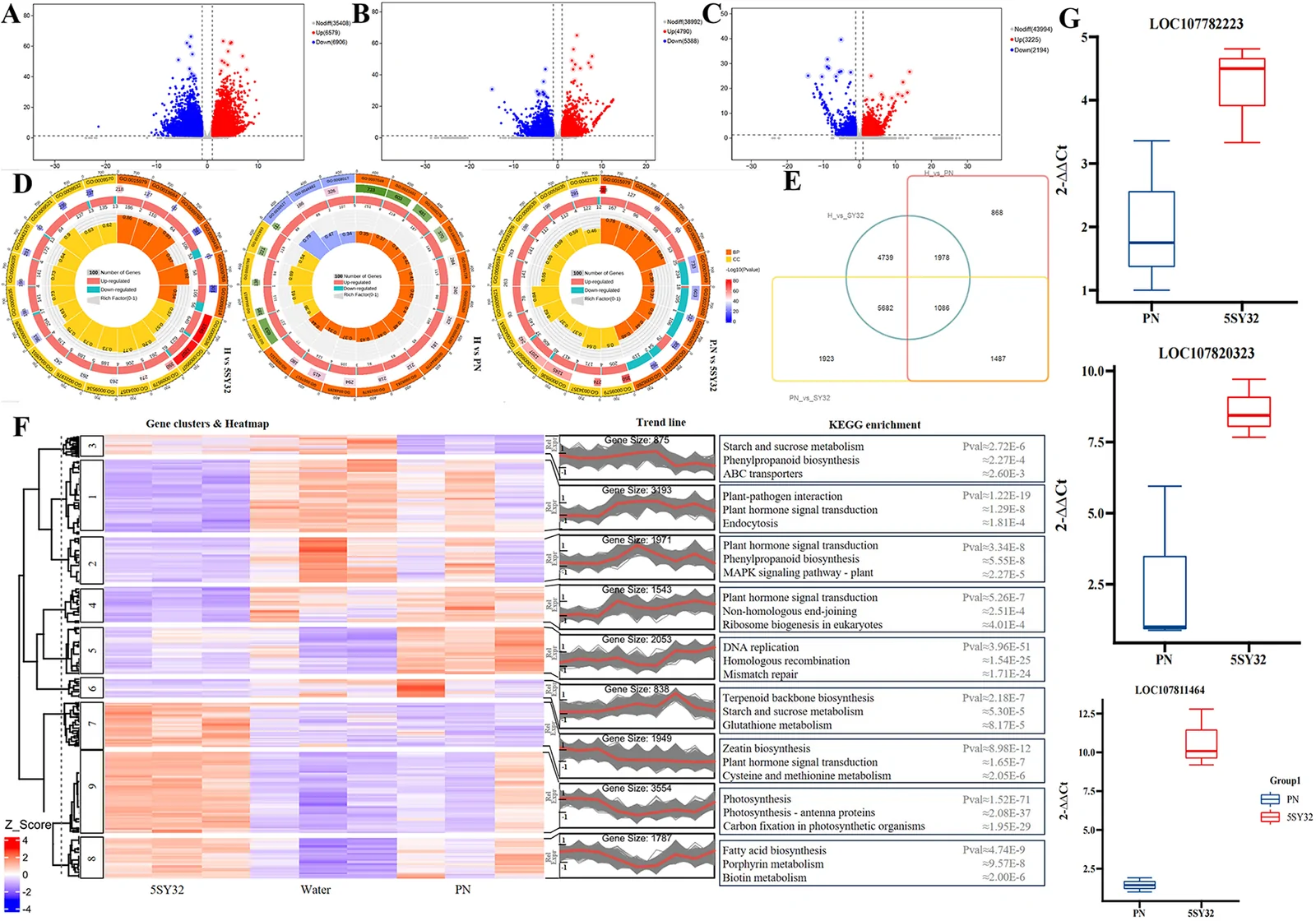
Transcriptomic profiling of tobacco plants treated with 5SY32. **(A)** Volcano plot showing differentially expressed genes (DEGs) between the water control (H_2_O) and strain 5SY32 treatment. **(B)** Volcano plot showing DEGs between the pathogen control (PN) and strain 5SY32 treatment. **(C)** Volcano plot showing DEGs between the pathogen control (PN) and water control (H_2_O). Red dots indicate significantly upregulated genes, blue dots indicate significantly downregulated genes, and gray dots indicate genes with no significant difference. **(D)** GO enrichment analysis of DEGs in the strain 5SY32 treatment group, categorized into Biological Process (BP), Cellular Component (CC), and Molecular Function (MF). **(E)** Venn diagram showing the overlap of DEGs in the strain 5SY32 treatment group compared to the water control (H_2_O) and pathogen control (PN). **(F)** Trend clustering and heatmap of common DEGs across all treatment groups, presenting 9 expression clusters with their representative trend lines and enriched KEGG pathways. **(G)** qPCR validation results of three representative DEGs involved in defense and secondary metabolism. Bar graphs represent the mean ± standard deviation (SD) of three biological replicates.

GO enrichment analysis revealed the treatment-specific functional classification of DEGs under different treatments. In the W vs. M comparison, the up-regulated DEGs were mainly enriched in a large number of chloroplast-related cell component entries, among which the representative structures included chloroplast matrix, thylakoid membrane, grana thylakoid, chloroplast inner membrane and outer membrane and other key photosynthetic organelle structures. At the same time, the enrichment results of biological processes focused on photosynthesis-related pathways, covering core physiological processes such as light reaction, light energy capture, chlorophyll biosynthesis and photosynthetic phosphorylation. These findings indicated that 5SY32 improved the photosynthetic capacity and energy acquisition of tobacco under pathogen-free conditions. This transcriptional signature was consistent with phenotypic variations, including elevated chlorophyll content, enlarged leaf area, and increased biomass in 5SY32-inoculated tobacco plants. Collectively, these results suggested that 5SY32 facilitates plant growth by reinforcing photosynthesis to provide sufficient energy reserves. In the P vs. M comparison, upregulated DEGs were mainly enriched in defense responses, oxidative stress responses, hormone signaling pathways, and cell wall organization. Specifically, genes associated with reactive oxygen species (ROS) scavenging (e.g., *POD* and *SOD*) and cell wall metabolism were significantly upregulated, demonstrating that 5SY32 potentiates plant immune responses and resistance-related pathways under pathogen stress. For the W vs. P comparison, DEGs were enriched in defense-, oxidative stress-, and hormone signaling-related terms, whereas the enrichment of photosynthesis and carbon metabolism pathways was diminished. This metabolic transition indicated that pathogen infection drives plants to reallocate metabolic resources from growth-oriented processes to defense responses (Figure 6D). Furthermore, a total of 1,086 common DEGs were identified across all comparison groups (Figure 6E).

Trend clustering analysis was performed to characterize the transcriptomic expression patterns modulated by 5SY32. All qualified DEGs were categorized into nine clusters (C1–C9) after data normalization. Genes clustered in C3, C7, C8, and C9 were significantly upregulated following 5SY32 treatment. Functional enrichment showed that these upregulated genes were primarily involved in energy metabolism (oxidative phosphorylation and carbohydrate metabolism), redox homeostasis, signal transduction (MAPK and JA signaling), cell wall metabolism, and antioxidant responses. Several key resistance-related genes were continuously and highly expressed in these clusters, suggesting the activation of defense-associated regulatory networks by 5SY32.

For instance, the signal transduction genes *JAR1, ERF1L8*, and *PP2C*, which are involved in JA-Ile biosynthesis, JA/ET-mediated defense activation, and ABA signaling, respectively, were markedly upregulated in C3, C7, C8, and C9, suggesting the coordinated activation of multiple hormone signaling pathways associated with induced systemic resistance. Moreover, multiple *POD* and *SOD* genes related to redox balance exhibited a sustained upward expression trend. These results indicated that 5SY32 effectively activates plant defense pathways and coordinates energy metabolism and physiological regulation in tobacco (Figure 6F).

KEGG enrichment analysis further verified the differential expression of key signaling genes. Consistent with the above results, *JAR1, ERF1L8*, and *PP2C* were significantly upregulated in 5SY32-treated plants. As a vital enzyme in the JA signaling cascade, the upregulation of *JAR1* indicated the activation of JA-mediated defense signaling for systemic resistance induction. The increased expression of *ERF1L8* enhanced the perception of microbe-associated molecular patterns (MAMPs), thereby facilitating pattern-triggered immunity (PTI). Additionally, the transcriptional alteration of *PP2C* reflected the dynamic regulatory balance of the abscisic acid (ABA) signaling pathway during defense activation. The upregulation of *PP2C* potentially suppresses ABA signaling, thereby indirectly enhancing the dominance of JA and ET pathways to strengthen pathogen resistance (Figure 6F). Collectively, these data demonstrated that 5SY32 enhances tobacco systemic resistance and environmental adaptability via a multi-layered defense activation mechanism. Furthermore, the expression trends of three randomly selected DEGs were validated by qPCR, which was highly consistent with the transcriptome sequencing results (Figure 6G).

### 3.8 A mechanistic model of 5SY32

Based on the agronomic trait assays and transcriptomic data analysis concerning the growth-promoting and disease-suppressive effects of 5SY32, a putative mechanistic model for 5SY32 was constructed (Figure 7).

**Figure 7:**
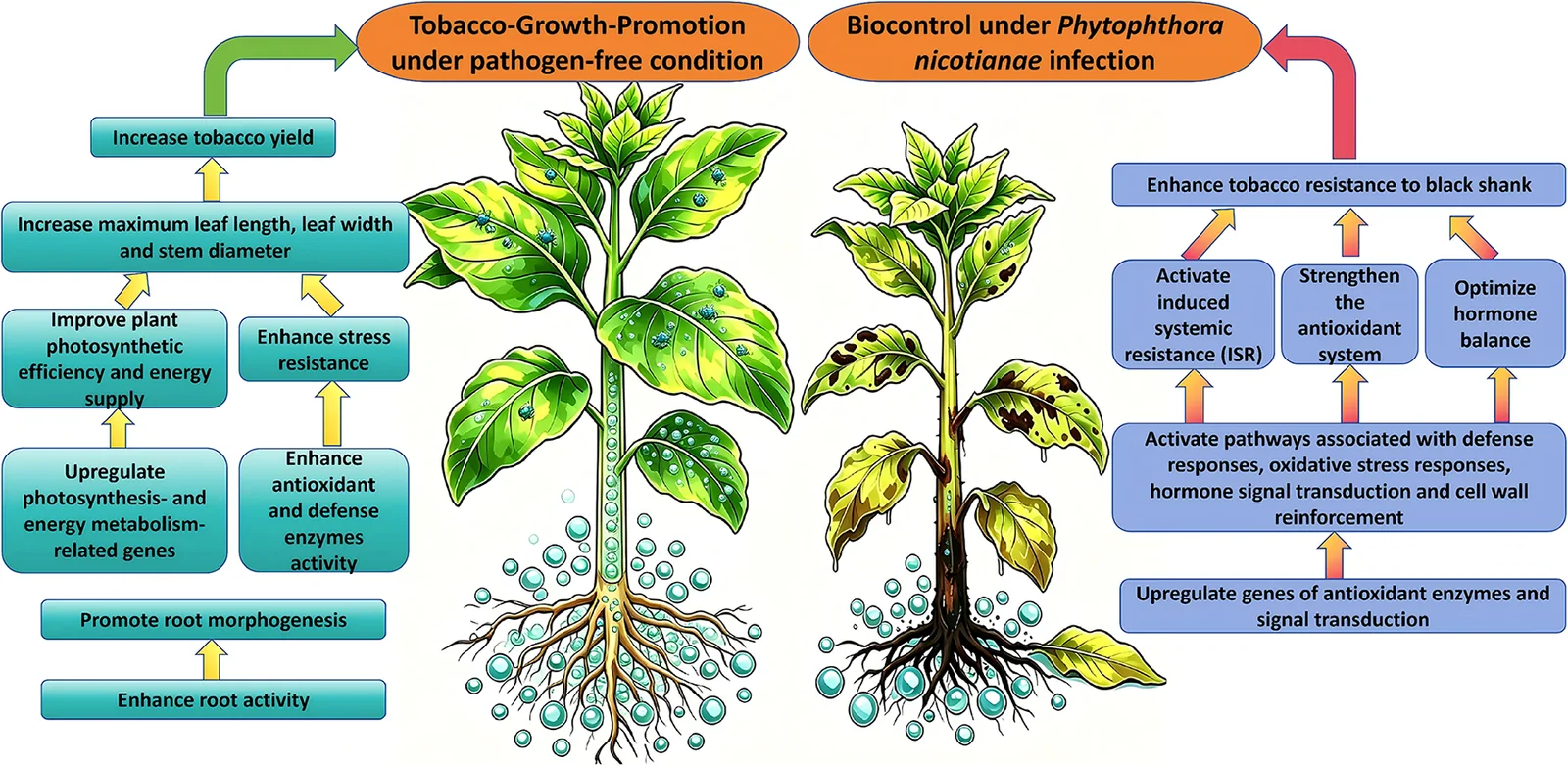
The mechanistic model of 5SY32.

5SY32 can successfully colonize tobacco plants. In the absence of P. nicotianae infection, root irrigation with 5SY32 significantly enhances root activity and promotes root morphogenesis of tobacco plants. Moreover, 5SY32 treatment markedly remodels the tobacco transcriptome, mainly manifested as the upregulated expression of genes related to photosynthesis and energy metabolism, which improves the photosynthetic efficiency and energy supply of tobacco plants. Meanwhile, the activities of antioxidant and defense-related enzymes, including POD, SOD, CAT, APX, PAL, and PPO, are significantly elevated, thereby strengthening the stress tolerance of tobacco plants. These positive regulatory effects increase the maximum leaf length, leaf width, and stem diameter of tobacco plants, and ultimately improve tobacco leaf yield.

Under the infection stress of *P. nicotianae*, root irrigation with 5SY32 significantly upregulates the transcription of antioxidant genes (e.g., *POD* and *SOD*) and key signal transduction genes (e.g., *JAR1, ELF1L8*, and *PP2C*). This activation triggers multiple downstream pathways, including plant defense responses, oxidative stress responses, hormone signal transduction, and cell wall reinforcement. Furthermore, 5SY32 activates the JA/ET-dependent induced systemic resistance (ISR) pathway, strengthens the antioxidant system, and optimizes endogenous hormone balance in tobacco. These molecular and physiological changes effectively induce tobacco resistance to *P. nicotianae*, achieving efficient biological control against TBS.

## 4 Discussion

Xibu Zhongyan 100, the leading cultivar in Luoyang tobacco production over the past decade, was selected for this study due to its regional representativeness and agronomic value. Field observations have consistently confirmed its susceptibility to soil-borne diseases-a fact well recognized in the industry, yet lacking formal publication. In our trials, this cultivar showed a DI of >70% under greenhouse conditions and > 40% in the field. Per the Chinese standard GB/T 23224-2008, these values correspond to S (greenhouse) and MS (field) classifications; we therefore consider it susceptible for this investigation. Currently, seed coating with 62.5 g/L metalaxyl-M and fludioxonil (Syngenta Nantong) is the standard chemical measure against root and stem diseases. However, its sustained use poses risks of resistance and pollution, prompting a search for biological alternatives. Accordingly, this study aimed to isolate antagonistic endophytic bacteria from tobacco against *P. nicotianae*, evaluate their black shank control efficacy, and explore their functional mechanisms, thereby contributing microbial resources and mechanistic insights to sustainable disease management.

To address this need, we systematically evaluated the colonization capability, growth-promoting potential, and biocontrol efficacy of *M. foliorum* 5SY32 in tobacco via greenhouse experiments, field trials, and transcriptomic profiling. Multiple lines of phenotypic, physiological, and molecular evidence consistently demonstrated that 5SY32 remarkably facilitates tobacco growth and development while suppressing TBS. Collectively, 5SY32 is characterized as a promising multifunctional endophytic bacterium with dual growth-promoting and disease-resistant capacities.

A hallmark trait of endophytic bacteria is their capacity to penetrate the plant epidermal barrier and form stable symbiotic associations within host tissues without triggering immune rejection (Lopez Del Egido et al., 2017; Frank et al., 2017). Colonization assays revealed that 5SY32 efficiently colonized the root cortex, stem vascular vessels, and intercellular spaces of tobacco leaves, reflecting high host compatibility of this strain. Previous studies have documented that *Microbacterium* endophytes can evade host immune recognition by modifying surface components (e.g., polysaccharides, lipopolysaccharides, and extracellular polymeric substances), thereby mitigating MAMP-triggered immune responses (Christian et al., 2015). Accordingly, 5SY32 may adopt a similar immune evasion strategy to achieve low-immunogenic endophytic colonization, which lays a foundation for sustained host-microbe interactions.

At the physiological level, 5SY32 markedly improved root architecture and leaf development in tobacco. It is well established that endophytes modulate plant cell division and elongation by regulating the biosynthesis, transport, and signal transduction of phytohormones, among which IAA, cytokinin (CTK), and gibberellin (GA) serve as core regulatory signals (Erbs and Newman, 2012; Ismail et al., 2021; Chen et al., 2022). The phenotypic alterations observed in this study, including enhanced root branching, expanded leaf size, and elevated chlorophyll and nitrogen contents, were consistent with plant growth traits modulated by the synergistic interplay between IAA and CTK. Transcriptomic analyses further provided molecular insights into these physiological variations. KEGG enrichment analysis indicated that DEGs were significantly enriched in the plant hormone signal transduction pathway, covering multiple signaling branches associated with IAA, CTK, and GA. Moreover, numerous DEGs related to auxin response, cell division modulation, and GA signaling regulation exhibited prominent expression changes. These findings confirm that 5SY32 remodels the host phytohormone signaling network at the transcriptional level. Therefore, the growth-promoting performance of 5SY32 is governed by the coordinated activation of multiple phytohormone signaling pathways rather than a single hormone, which optimizes plant growth, development, and physiological homeostasis. In summary, 5SY32 acts as a potent hormonal modulator to boost tobacco growth.

Transcriptomic data also showed that upregulated genes in the 5SY32-treated tobacco plants were predominantly enriched in photosynthesis-related biological processes and chloroplast-associated cellular components, including light-reaction modules. This molecular evidence indicates that 5SY32 enhances the photosynthetic capacity and energy acquisition efficiency of tobacco. Notably, this transcriptional signature was highly consistent with the phenotypic improvements in chlorophyll accumulation, biomass production, and plant yield. Collectively, these results suggest that the growth-promoting effects of 5SY32 depend not only on morphological optimization but also on the reinforcement of photosynthetic metabolism, which provides sufficient energy for plant sustained growth.

Compared with the control group, 5SY32 treatment significantly increased the activities of defense-related enzymes (e.g., PAL, PPO, POD, SOD, and CAT), indicating activation of the tobacco defense system. However, the induction of defense-related enzymes alone is insufficient to distinguish between systemic acquired resistance (SAR) and induced systemic resistance (ISR). Transcriptomic profiling revealed that key genes involved in JA and ET signaling pathways (e.g., *LOX2, AOS*, and *ERF1*) were markedly upregulated, whereas salicylic acid (SA)-related genes showed negligible transcriptional changes. Moreover, additional JA/ET signaling components, including *JAR1* and *ERF1L8*, were also significantly upregulated after 5SY32 inoculation. Such a transcriptional pattern, characterized by activation of JA/ET signaling with minimal changes in SA-responsive genes, is widely recognized as a hallmark of ISR rather than SAR. These results demonstrate that the strain-triggered defense response is predominantly mediated by JA/ET-dependent signaling cascades, instead of SA-dependent systemic acquired resistance (SAR). Generally, ISR activation is attributed to the specific recognition of endophyte-derived signaling molecules by host plants, such as lipopeptides, small peptides, and volatile organic compounds (VOCs) (Chen et al., 2024; Tyagi et al., 2018; Hossain and Chung, 2019). Hence, 5SY32 may activate host defense systems via specific secreted signaling molecules.

Differential expression of *JAR1, ERF1L8*, and *PP2C* further strengthened the conclusion that 5SY32 activates JA/ET-dependent ISR while simultaneously coordinating ABA-mediated stress responses. *JAR1* encodes the enzyme responsible for conjugating jasmonic acid with isoleucine to produce JA-Ile, the bioactive form of jasmonate required for activating JA signaling. Its upregulation therefore suggests enhanced JA-dependent defense signaling and supports the induction of ISR by 5SY32 (Choudhary and Johri, 2009). As a member of the ethylene response factor family, *ERF1L8* acts downstream of JA and ET signaling and serves as an important transcriptional integrator of these two pathways, thereby activating defense-related genes and strengthening resistance against pathogen invasion (Kazan and Manners, 2008). Additionally, *PP2C* is a key regulatory component of the ABA signaling pathway, and its differential expression suggests dynamic modulation of ABA-mediated stress responses. Such regulation may contribute to balancing immune activation with plant growth, thereby reducing the fitness costs associated with constitutive defense responses (Li et al., 2022; Khan, 2025). Furthermore, defense-related metabolic pathways (e.g., phenylpropanoid metabolism, flavonoid biosynthesis, and lignin biosynthesis) were prominently enhanced. The secondary metabolites produced by these pathways can directly inhibit pathogen proliferation and reinforce plant cell walls, forming integrated physical and chemical defensive barriers against pathogens (Ma et al., 2009; Ramaroson et al., 2022; Ortiz and Sansinenea, 2023). The transcriptomic evidence described above is also consistent with the characteristic features of ISR rather than SAR. Unlike SAR, which is typically associated with SA accumulation and the activation of SA-responsive defense genes, endophyte-triggered ISR primarily depends on JA/ET signaling and maintains plants in a primed immune state with relatively low fitness costs. Notably, endophyte-triggered ISR is distinct from pathogen-induced SAR, as ISR maintains a primed immune state without excessive energy consumption (Yadav et al., 2020). 5SY32-induced resistance shared this typical feature: defense enzyme activities were moderately elevated under pathogen-free conditions without growth inhibition, and plants could rapidly launch amplified defense responses upon pathogen infection. Collectively, 5SY32 balances the growth-defense trade-off in tobacco via the synergistic regulation of JA/ET and ABA signaling pathways. Moreover, several ABA-related genes (e.g., *PYR/PYL* and *PP2C*) were differentially expressed after 5SY32 inoculation, suggesting that ABA signaling contributes to fine-tuning defense activation while maintaining normal plant growth. Such multi-signal integration capability is a unique characteristic that differentiates endophytes from rhizosphere bacteria, accounting for their dual growth-promoting and biocontrol functions.

Endophytic bacteria can establish persistent symbiosis with host plants and sustainably improve plant resistance and ecological adaptability (Radhakrishnan et al., 2017; Wani et al., 2015). 5SY32 maintained stable growth-promoting and biocontrol performance under both greenhouse and field conditions, demonstrating excellent ecological stability. Unlike conventional rhizosphere plant growth-promoting bacteria, endophytes are less susceptible to environmental fluctuations and directly modulate the internal signaling networks of host plants, thereby demonstrating more stable efficacy in complex agricultural ecosystems. Consistent with the above results, 5SY32 enhances tobacco health mainly by remodeling host signaling pathways rather than relying on direct microbial antagonism. This superior trait endows 5SY32 with great application potential in biological control and biofertilizer development. One limitation of this study, the colonization of strain 5SY32 in tobacco tissues was evaluated using the plate re-isolation method combined with colony PCR verification. While this approach confirmed the recoverability and identity of the inoculated strain, it does not fully capture the actual in-plant colonization status, as it only detects culturable cells and cannot distinguish epiphytic from endophytic populations. Future studies employing GFP tagging, immunofluorescence, or fluorescence *in situ* hybridization (FISH) are warranted to provide direct visualization of strain 5SY32 within host tissues. Another limitation of the present study is that an autoclaved 5SY32 treatment was not included as an additional negative control. Although an LB medium control was incorporated throughout the experiments to minimize the potential influence of residual culture medium nutrients, we acknowledge that a heat-killed bacterial control would further distinguish the effects of viable bacterial colonization from those of bacterial cell components. In our preliminary experiments, autoclaving markedly reduced the antagonistic activity of strain 5SY32, likely because heat treatment degraded heat-sensitive bioactive metabolites. Therefore, the present study focused primarily on evaluating the biological functions of viable 5SY32. Future studies incorporating heat-killed bacterial controls will further clarify the relative contributions of living cells and bacterial-derived components to induced disease resistance and rhizosphere microbial modulation. Future multi-omics studies combining genomics and metabolomics will further identify its key signaling molecules and functional gene clusters, providing a solid molecular foundation for the industrial application of this beneficial endophyte.

In field trials, 5SY32 exhibited significantly superior control efficacy against TBS compared with the registered commercial agent, 108 CFU/g *B. subtilis* microcapsule granule. Beyond variables including formulation type and application strategy, differences in biocontrol taxa and microbial origin constituted the primary drivers for this discrepancy. Relative to well-studied *B. subtilis, M. foliorum* remains an under-explored taxon for biological control and represents an underexploited resource for future biopesticide development. Strain origin, in particular, underpinned the prominent field performance of 5SY32. Isolated from surface-sterilized healthy tobacco tissues, 5SY32 acts as a true plant endophyte, which confers intrinsic advantages in host tissue colonization capacity and efficient activation of tobacco immune responses, collectively contributing to its high field efficacy against *P. nicotianae* (Ali et al., 2024). By contrast, commercially available biocontrol agents such as the *B. subtilis* preparation used in this study are mostly derived from non-tobacco niches; such exogenous strains frequently display inferior in-planta colonization and weaker capacity to trigger host defense responses compared with host-adapted endophytic isolates (Liu et al., 2020).

Despite the promising application potential of strain 5SY32, several bottlenecks remain to be addressed prior to its industrial development and field deployment. First, formulation development represents an urgent priority for 5SY32. It remains unclear which formulation type-including wettable powder, granule, water-dispersible granule, microcapsule granule, and suspension concentrate-can maximize its plant-growth-promoting and disease-suppressive performance under field conditions. Second, multi-year and multi-location field trials are indispensable. The field assays in the present study were only conducted across two sites within a single year, which is insufficient to robustly validate its practical application potential. Therefore, its consistent field performance requires further verification. Third, all experiments herein were performed using only one tobacco cultivar, whereas diverse tobacco cultivars are cultivated across different tobacco-growing regions. Whether 5SY32 can retain favorable efficacy on other tobacco cultivars still needs further investigation. Resolving the above-mentioned issues will consolidate the practical value of 5SY32 for green management of tobacco diseases, and relevant research is currently underway.

## 5 Conclusion

As a tobacco endophytic bacterium, *M. foliorum* 5SY32 colonizes tobacco tissues. It exerts synergistic growth-promoting and disease-resistant effects by activating the JA/ET-dependent ISR pathway, enhancing the antioxidant defense system, and regulating endogenous hormone homeostasis. This study not only broadens the mechanistic framework for the functional exploration of endophytic bacteria but also provides promising microbial resources and an innovative biological control strategy against TBS.

## Data Availability

The data presented in the study are deposited in the Genome Sequence Archive (GSA) repository of the National Genomics Data Center, China National Center for Bioinformation / Beijing Institute of Genomics, Chinese Academy of Sciences, accession number CRA049801; the data are publicly accessible at https://ngdc.cncb.ac.cn/gsa.
